# Disturbed Complement Receptor Expression Pattern of B Cells Is Enhanced by Toll-like Receptor CD180 Ligation in Diffuse Cutaneous Systemic Sclerosis

**DOI:** 10.3390/ijms25179230

**Published:** 2024-08-26

**Authors:** Szabina Erdő-Bonyár, Judit Rapp, Rovéna Subicz, Katalin Böröcz, Dávid Szinger, Kristóf Filipánits, Tünde Minier, Gábor Kumánovics, László Czirják, Tímea Berki, Diána Simon

**Affiliations:** 1 Department of Immunology and Biotechnology, Clinical Center, University of Pécs Medical School, H-7624 Pécs, Hungary; erdo-bonyar.szabina@pte.hu (S.E.-B.); subicz.rovena@pte.hu (R.S.); borocz.katalin@pte.hu (K.B.); szinger.david@pte.hu (D.S.); berki.timea@pte.hu (T.B.); simon.diana@pte.hu (D.S.); 2 Department of Rheumatology and Immunology, Clinical Center, University of Pécs Medical School, H-7632 Pécs, Hungary; filipanits.kristof@pte.hu (K.F.); minier.tunde@pte.hu (T.M.); kumanovics.gabor@pte.hu (G.K.); czirjak.laszlo@pte.hu (L.C.)

**Keywords:** CD180, systemic sclerosis, complement receptors, Toll-like receptors, natural autoantibodies, B cells, C3, anti-Scl-70 autoantibodies

## Abstract

Autoantibody production is a hallmark of systemic sclerosis (SSc) and the most extensively studied role of B cells in the pathogenesis of the disease. However, the potential involvement of innate immune molecules in B-cell dysfunction in SSc is less understood. B-cell activation is an early event in the pathogenesis of SSc and is influenced by complement receptors (CRs) and Toll-like receptors (TLRs), shaping antibody responses. CR2 and CR1 modulate B-cell activation, and the roles of CR3 and CR4 are associated with autoimmune conditions. We investigated the expression of CRs in B cells from patients with the more severe form of the disease, diffuse cutaneous SSc (dcSSc), and the effect of TLR CD180 ligation on their expression. We found no significant difference in the basal expression of CD21 and CD11c in B cells between dcSSc and healthy controls (HCs). However, reduced basal CD11b expression in B cells in dcSSc compared to HCs, accompanied by a decrease in CD35 and an increase in CD11c expression following CD180 ligation may promote plasma cell formation and autoantibody production. Additionally, we searched for correlations between dcSSc-associated anti-DNA topoisomerase I (Scl-70) autoantibody, anti-citrate synthase (CS) natural autoantibody and complement component 3 (C3) levels and found a negative correlation between C3 and anti-CS autoantibody in dcSSc but not in HCs, supporting the hypothesis that natural autoantibodies could activate the complement system contributing to tissue injury in SSc.

## 1. Introduction

Systemic sclerosis (SSc) is a multifactorial, immune-mediated rheumatic disease, characterized by vascular damage, subsequent fibrosis, autoimmune abnormalities. Diffuse cutaneous SSc (dcSSc) is the more severe form of the disease [1]. Recent evidence highlights the role of B-cell activation at the beginning of disease development, as B-cell infiltration is detectable in cutaneous areas prior to the onset of fibrosis [2]. The abnormal activation of B cells involves B-cell receptors (BCRs) and germline-encoded coreceptors, such as complement receptors (CRs) and Toll-like receptors (TLRs). Abnormal CR signaling can contribute to B-cell differentiation towards an autoreactive phenotype, with well-established roles for CD21 and CD35, while the functions of CD11b and CD11c in this process are increasingly recognized, as they can promote the development of autoantibody-producing cells [3,4,5,6,7].

The interplay between CRs and TLRs in regulating B-cell functions is well described [3], and recent evidence implicates the contribution of TLR activation to B-cell biology, particularly in shaping antibody responses [8]. CD180 is an orphan TLR receptor expressed on human B cells and modulates B-cell functions [8]. CD180 was first described as a TLR4 homologue influencing its effects [9], and TLR4 is strongly linked to fibrosis in SSc but not directly to B-cell dysfunctions [10]. However, our previous results indicate the potential importance of CD180-mediated B cell-functions in SSc [11,12]. We showed that the CD180 ligation of B cells resulted in a more pronounced activation of natural autoantibody-producing marginal zone-like non-switched memory B cells in SSc than in healthy controls (HC) [11], and stimulation via CD180 enhanced natural autoantibody production [13]. Natural autoantibodies are mainly IgM molecules present in the sera in healthy individuals and patients with autoimmune disease prior to antigen exposure [14]. Their target antigens are biologically conserved structures, and natural autoantibodies recognizing injured cell surfaces facilitate the clearance of damaged cells but can also activate complement within the damaged tissue [15]. The initial manifestation of SSc is related to vascular abnormalities and repeated ischemia-reperfusion events [16]. The neoepitopes revealed during tissue ischemic injury are recognized by IgM natural autoantibodies, resulting in activation of the complement cascade [17]. The localized activation of the complement system in SSc, with abnormal membrane attack complex (MAC) deposition on the endothelium, has already been reported [18,19]. The pathogenic effect of SSc-specific autoantibody-containing immune complexes, including anti-DNA topoisomerase I (Scl-70) autoantibody, on endothelial cells has been described, suggesting that these immune complexes trigger activation of the endothelium and disrupt the normal function of TLRs [20]. Therefore, the purpose of this study was to investigate the expression of CRs and the effects of CD180 ligation on them in B cells from patients with dcSSc. A further aim was to correlate serum levels of natural and SSc-specific autoantibodies with the central complement component 3 (C3) to assess whether the interaction between TLRs, complement components and B cells could play a role in the pathogenesis of SSc.

## 2. Results

### 2.1. CD21 mRNA Expression in B Cells in dcSSc Is Similar to HC

**Figure 1:**
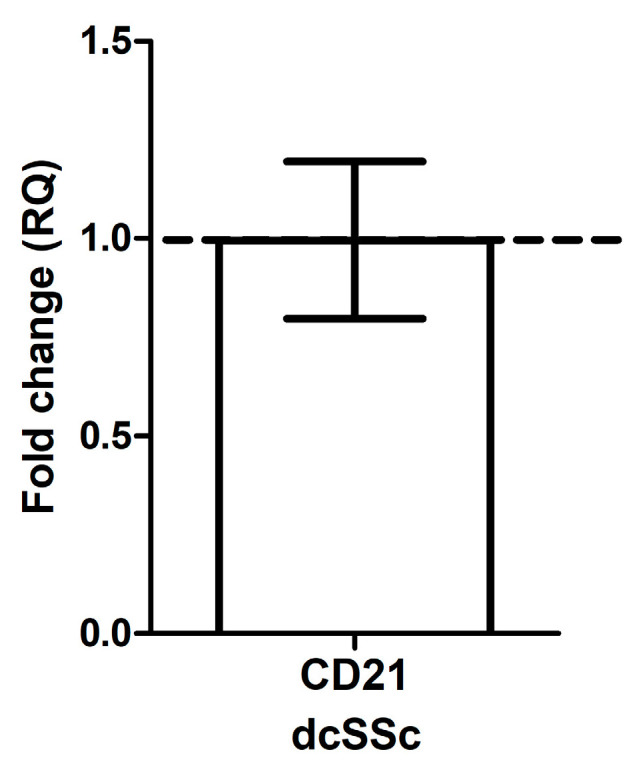
The mRNA expression of CD21 in purified B cells from diffuse cutaneous systemic sclerosis (dcSSc) patients (*n* = 4) and healthy controls (HC) (*n* = 4). Gene expression of dcSSc patients was normalized to HCs, whose expression levels are indicated by the horizontal line (value 1). Changes in gene expression are shown as relative quantification (RQ) values. Data are shown as mean ± standard error of the mean (SEM).

CD21 is part of the BCR complex, and its reduced expression was observed in B cells from patients with systemic lupus erythematosus (SLE) [3]. Therefore, we first analyzed the mRNA expression of CD21 in purified B cells from patients with dcSSc and HCs and found no significant difference between dcSSc and HC (Figure 1).

### 2.2. Decreased CD35 Expression on B Cells in dcSSc

**Figure 2:**
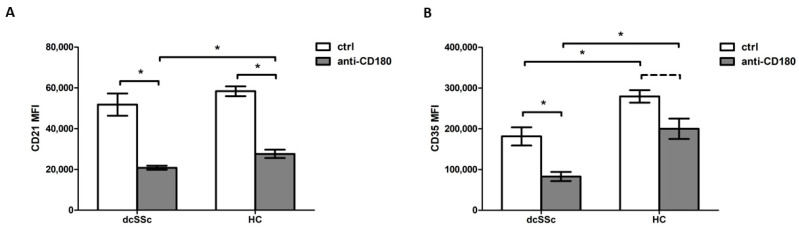
Protein expression of CD21 and CD35 on the B cells in dcSSc and HCs. The mean fluorescence intensity (MFI) of CD21 (**A**) and CD35 (**B**) of B cells was measured by flow cytometry in dcSSc (*n* = 4) and HCs (*n* = 4) after anti-CD180 antibody stimulation (anti-CD180) or left unstimulated (ctrl). The solid lines show significant differences (* *p* < 0.05), while the dashed line indicates tendency (*p* < 0.1). Data are presented as means ± SEM.

Since the expression levels of CD21 mRNA did not differ between dcSSc and HC B cells, we examined its protein expression level. No significant difference in CD21 protein levels was found between dcSSc and HC B cells (Figure 2A). As CD35 is known to be an important inhibitory complement receptor in BCR-mediated B-cell function [4], we then investigated the protein expression of CD35 in dcSSc and HC B cells. A significantly decreased level of CD35 was observed in the B cells of dcSSc patients compared to HCs (Figure 2B).

Since the crosstalk between CRs and TLRs in the regulation of B-cell functions has already been reported [3,8], we investigated the effect of activation via the TLR homologue CD180 on the expression of CD21 and CD35 on B cells in dcSSc and HCs. The anti-CD180 antibody treatment significantly diminished CD21 expression in both dcSSc and HC B cells; moreover, significantly lower CD21 expression levels were found in dcSSc compared to HC upon stimulation (Figure 2A). Treatment with an anti-CD180 antibody significantly decreased CD35 expression in B cells in dcSSc and showed a tendency to decrease in HC; thus, the reduced CD35 expression in dcSSc B cells observed under unstimulated conditions was maintained after stimulation (Figure 2B).

### 2.3. CD180 Ligation Has Opposite Effects on CD11b and CD11c Expression in B Cells

**Figure 3:**
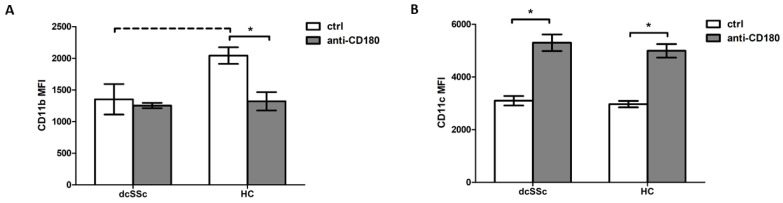
Expression of CD11b and CD11c on the B cells in dcSSc and HCs. The MFI of CD11b (**A**) and CD11c (**B**) of B cells was measured by flow cytometry in dcSSc (*n* = 4) and HCs (*n* = 4) after stimulation with the anti-CD180 antibody (anti-CD180) or left unstimulated (ctrl). The solid lines show significant differences (* *p* < 0.05), while the dashed line indicates tendency (*p* < 0.1). Data are presented as means ± SEM.

CD11b and CD11c are recently discovered and less studied CRs, but their possible contribution to autoimmune diseases has been suggested [3]. The expression of CD11b tended to be lower in the B cells of dcSSc patients compared to HCs (Figure 3A). No significant differences in CD11c expression levels were found between the dcSSc and HC B cells (Figure 3B). Stimulation via CD180 reduced CD11b expression in B cells only in the HCs, but not in the dcSSc patients; thus, stimulation with anti-CD180 antibody in the HCs shifted CD11b expression to the levels observed in dcSSc (Figure 3A). Stimulation through CD180 significantly increased CD11c expression levels in both the dcSSc and HC B cells, but, similar to the unstimulated conditions, no significant difference in CD11c expression was found between dcSSc and the HCs after stimulation (Figure 3B).

### 2.4. Natural IgM Autoantibodies against CS Negatively Correlate with C3 Serum Levels in dcSSc

**Figure 4:**
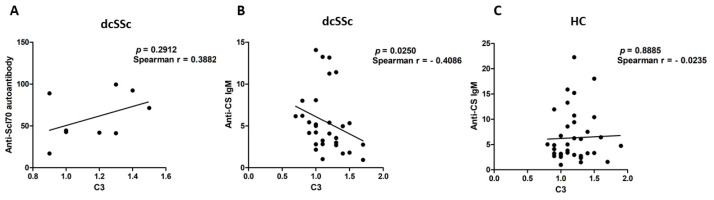
Association between serum levels of autoantibodies and C3 in dcSSc and HCs. Correlation between anti-Scl-70 autoantibody levels and C3 serum levels in dcSSc (*n* = 9) (**A**); correlation between anti-CS IgM natural autoantibody and C3 serum levels in dcSSc (*n* = 30) (**B**) and HC (*n* = 38) (**C**). Spearman’s correlation analysis was performed to determine the level of statistical significance. Correlation coefficient (r) and *p* value are indicated.

The presence of pathological autoantibodies is a feature of dcSSc, and immune complexes containing dcSSc-specific autoantibodies were shown to be associated with endothelial cell alterations in SSc [20]. Therefore, we examined the relationship between pathological IgG autoantibodies and C3 levels but found no significant correlation between the dcSSc-specific pathological autoantibody levels, anti-Scl-70 autoantibody levels, and serum C3 levels in dcSSc (*p* = 0.2912, Spearman r = 0.3882) (Figure 4A).

Furthermore, as it has already been suggested that natural IgM autoantibodies can also activate the complement system [17], we looked for a possible correlation between serum levels of natural IgM autoantibody levels against CS and C3 in dcSSc and the HCs. A significant negative correlation was observed between anti-CS IgM autoantibody levels and C3 levels in dcSSc (*p* = 0.0250, Spearmen r = −0.4086) (Figure 4B). No significant correlation was found between anti-CS IgM autoantibodies and C3 levels in HCs (*p* = 0.8885, Spearman r = −0.0235) (Figure 4C). In addition, we observed a positive correlation between C3 serum levels and inflammatory laboratory parameters such as C-reactive protein (CRP) (*p* = 0.0300, Spearman r = 0.3725), the erythrocyte sedimentation rate (ESR) (*p* = 0.0085, Spearman r = 0.4442) and white blood cell count (*p* = 0.0407, Spearman r = 0.3429) in dcSSc.

## 3. Discussion

B-cell activation has been reported to be an early event in the pathogenesis of SSc. While the B-cell function is primarily governed by BCR signaling, germline-encoded receptors, such as CRs and TLRs, also significantly influence its overall activity. CD21 has been identified as a coreceptor of BCRs, with an important role in lowering the threshold for BCR stimulation [21]. In addition, a prominent role for CD21 in the maintenance of peripheral B-cell tolerance has been suggested, and diminished CD21 expression by B cells has been observed in SLE [3]. However, according to our results, its expression at the mRNA level and protein level was comparable in B cells in dcSSc and the HCs. The co-ligation of CD35 with BCR can exhibit various inhibitory functions on B cells, including impaired proliferation, activation and antibody production [4]. Investigating the CD35 expression of B cells, we found a significantly decreased level in dcSSc compared to the HCs. Our findings on CD21 and CD35 are in alignment with Soto’s observations [22]. Limited research has been carried out on the involvement of the integrin-type CRs, CD11b and CD11c in SSc, and their roles in autoimmunity are not fully understood, but there is growing recognition of their involvement in supporting autoreactive B cell formation [3]. Although CD11b and CD11c are closely related, as CD11c shares 63% sequence homology with CD11b [23], their different cellular distribution and ligand specificity result in distinct functional characteristics [24,25,26]. CD11c can promote B-cell differentiation into antibody-secreting cells [27], while the lack of CD11b on B cells favored the autoreactive B-cell phenotype and autoantibody production [7]. We found that the CD11b expression of B cells was significantly lower in dcSSc compared to the HCs, while CD11c, similarly to CD21, was expressed at the same level in B cells from dcSSc and the HCs. Complement and the TLR system are two essential players of innate immunity, and emerging literature suggests their interplay in autoimmune diseases, particularly in antibody responses [3,4]. In SLE, CD11b can act as a negative regulator of TLR signaling, where the lack of CD11b leads to increased autoantibody production [28]. Not only is TLR signaling affected, but BCR can also be negatively regulated by CD11b, promoting an autoreactive B-cell phenotype [29]. CD11c has been reported to promote plasma cell formation from memory B cells [27]. As autoantibody production is a hallmark of SSc [1], it is plausible to investigate TLR-mediated activation of B cells. Our previous data suggest that CD180 has a role in B-cell dysfunction in SSc; therefore, we investigated the effect of CD180 ligation on the expression of CRs. The B cells from patients with dcSSc mostly responded similar to those from the HCs; the expression of CD35 and CD21 was decreased, while the expression of CD11c was increased upon stimulation via CD180. However, the CD11b levels in B cells decreased after CD180 ligation only in the HCs, reaching levels similar to basal expression in the dcSSc B cells. The reduced basal CD11b expression of B cells in dcSSc compared to the HCs, along with the decrease in CD35 and increase in CD11c expression following B-cell activation via CD180 could favor the formation of autoreactive B cells and enhance autoantibody production.

Microvascular malfunctions are typical results of abnormal B-cell activation followed by autoantibody production [30]. Immune complexes containing SSc-specific antibodies might contribute to the SSc pathogenesis through a direct interaction with TLRs and the induction of endothelial cell activation [20]. Localized activation of the complement system in SSc through abnormal MAC deposition on the endothelium and decreased expression of complement regulatory factors were reported in skin lesions, which are known to induce endothelial cell apoptosis [18,19]. However, we found that anti-Scl-70 autoantibody and C3 levels were not correlated, suggesting that anti-Scl-70 autoantibodies could induce endothelial alterations via other mechanisms than the fixation and activation of the complement cascade, such as antibody-dependent cell-mediated cytotoxicity, which has already been described in SSc in the case of anti-endothelial cell autoantibodies [31,32,33].

We previously found that CD180 ligation augments the production of natural autoantibodies, which are capable of recognizing neoantigens during ischemic damage [17]. The initial manifestation of SSc is related to vascular abnormalities. Raynaud’s phenomenon is an early symptom in SSc, with subsequent reperfusion injury in the background [16]. The role of natural antibodies in ischemia-reperfusion injury is supported by pioneering studies demonstrating that reconstituting recombination-activating gene 1 (RAG-1) knockout mice, which lack mature B and T lymphocytes, with purified IgM from wild-type sera restores their susceptibility to ischemia-reperfusion injury [34]. Evidence from mice experiments using RAG-1 and complement component 4 knockout mice suggested the activation of the classical pathway in the process [35]. Studies investigating renal injuries indicate that reperfusion injury is mainly mediated by the alternative complement pathway [36]. The activation of the lectin pathway was also evaluated in SSc, and the serum levels of lectin pathway proteins were found to be associated with vascular dysfunction [37,38]. Regardless of which pathway is involved, once activated, the central component C3 gains enzymatic activity, resulting in the appearance of the C3b cleavage product. C3b cleavage fragments function as pivotal activators within the complement cascade, ultimately leading to the formation of the MAC. We found a negative correlation between serum levels of anti-CS IgM natural autoantibodies and C3 in dcSSc patients but not in the HCs, suggesting that the activation and consumption of C3 can be induced by natural autoantibodies in dcSSc and might contribute to vascular damage.

We can conclude that the expression pattern of CRs on the B cells of patients with dcSSc is different from that in the HCs, and the difference is further enhanced by stimulation via TLR CD180. As we have previously shown that the reduced CD180 expression in B cells in dcSSc may be the result of their activation via CD180 [13], we can propose that the effect of CD180 stimulation on CR expression is part of a complex mechanism resulting in the B-cell dysfunction observed in SSc. Furthermore, the found negative correlation between serum natural autoantibody and C3 levels in dcSSc may indicate the activation and consumption of C3 triggered by natural autoantibodies, which have been reported to potentially contribute to tissue damage. Consequently, we can hypothesize that the interaction between TLRs and complement components promoting plasma cell formation and autoantibody production could play a role in the pathogenesis of SSc.

## 4. Materials and Methods

### 4.1. Enrolled Individuals

Thirty-eight patients with diffuse cutaneous systemic sclerosis were chosen for this study. They all met the 2013 ACR/EULAR SSc classification criteria [39] and had a diffuse form of the disease, according to the classification system proposed by LeRoy et al. [40]. Physical examination and laboratory tests were performed at the time of patient recruitment. We excluded from this study patients with overlap syndromes, tumors and current infections. Mean (SD) disease duration was 7.1 (±5.6) years based on the date of the first non-Raynaud’s symptom, the mean (SD) age at enrollment was 53.34 (±13.6) years, the mean (SD) modified Rodnan skin score (mRSS) was 13.93 (±9.5) points. Frequent internal organ involvements were interstitial lung disease (78.9%), cardiac involvement (63.2%) and gastrointestinal involvement (52.6%). The detailed characteristics of the patients are described in Table 1. Age- and sex-matched healthy individuals (HCs) were used as controls (*n* = 46). All participants provided written informed consent for this study, following approval by the Hungarian National Ethics Committee (ETT TUKEB 47861-6/2018/EKU).

### 4.2. Peripheral Blood Mononuclear Cell Isolation and B-Cell Separation

Isolation of peripheral blood mononuclear cells (PBMCs) from peripheral blood samples of dcSSc patients (*n* = 8) and HCs (*n* = 8) was performed by density gradient centrifugation using Ficoll-Paque Plus (GE Healthcare, Chicago, IL, USA). The MACS B cell isolation kit II (Miltenyi Biotech, Bergisch Gladbach, Germany) was used for negative selection of B cells (n_SSc_ =4, n_HC_ =4). B-cell purity was above 95%.

### 4.3. RNA Isolation, cDNA Synthesis, and qPCR for the Evaluation of CD21 Expression

CD21 mRNA expression was determined in total B cells from dcSSc patients (*n* = 4) and HCs (*n* = 4), following RNA isolation and cDNA transcription, as previously described [11].

### 4.4. Flow Cytometric Analysis of CRs Expression

To investigate the expression CD35, CD21, CD11b and CD11c on B cell in dcSSc (*n* = 4) and HCs (*n* = 4), 5 × 10^5^ PBMCs were stimulated with Ultra-LEAF purified anti-human CD180 (RP105) antibody (Clone: MHR73-11) (Bio-Legend, San Diego, CA, USA) at 1 µg/mL (anti-CD180) or left unstimulated for 24 h at 37 °C. Following stimulation, PBMCs were labeled using the combination of the following monoclonal antibodies: anti-human CD19-Alexa Fluor 700 (SJ25C1, BioLegend, San Diego, CA, USA), anti-human CD35-PE (E11, BioLegend, San Diego, CA, USA), anti-human CD21-BV421 (B-ly4, BD Biosciences, Franklin Lakes, NJ, USA), anti-human CD11b-APC/Cyanine7 (ICRF44, BioLegend, San Diego, CA, USA) and anti-human CD11c-FITC (Bu15, BioLegend, San Diego, CA, USA), following the manufacturer’s protocols, as described earlier [12].

### 4.5. Natural Autoantibodies Measurement

The levels of anti-CS IgM autoantibodies were determined with an in-house ELISA, as previously described [41].

### 4.6. Statistical Analysis

The SPSS v. 27.0 statistics package (IBM, Armonk, NY, USA) was used to perform statistical analysis, with Student’s t-tests, Mann–Whitney U-test and Spearman’s correlation, where *p* values < 0.05 were regarded significant and *p* values < 0.1 were considered indicative of a tendency.

## Figures and Tables

**Table 1:** Patients’ characteristics.

Characteristics	dcSSc Patients (*n* = 38)
Age (years), mean (SD)	53.34 (13.6)
Gender (female), *n* (%)	31/38 (81.6%)
Disease duration ^1^ (years), mean (SD)	7.1 (5.6)
Organ involvement	
mRSS mean (SD)	13.93 (9.5)
Lung fibrosis ^2^, *n* (%)	30/38 (78.9%)
Cardiac involvement ^3^, *n* (%)	24/38 (63.2%)
Gastrointestinal involvement ^4^, *n* (%)	20/38 (52.6%)
Antibodies	
Anti-Scl-70+, *n* (%)	15/38 (39.5%)
Anti-RNA-polymerase III+, *n* (%)	7/38 (18.4%)

^1^ From the date of the first non-Raynaud’s symptom; ^2^ determined by detection of fibrosis with high resolution CT and/or decreased forced vital capacity (FVC < 80%); ^3^ identified by diastolic dysfunction or decreased left ventricular ejection fraction; ^4^ confirmed by barium swallow or esophago-gastroscopy.

## Data Availability

Data are contained within the article.
